# Irreducible Knee Dislocation With Vastus Medialis Muscle Interposition: A Case Report

**DOI:** 10.7759/cureus.33409

**Published:** 2023-01-05

**Authors:** Nicolae Angan, Boris Feghiu, Nicolae Gheorghiu, Valentin Feghiu, Muhammad Usaama Bahadoor

**Affiliations:** 1 Trauma and Orthopaedic Surgery, Letterkenny University Hospital, Letterkenny, IRL; 2 Trauma and Orthopaedic Surgery, Elias Emergency University Hospital, Bucharest, ROU; 3 Trauma and Orthopaedic Surgery, Territorial Medical Association, Chisinau, MDA

**Keywords:** posterolateral knee dislocation, complicated knee dislocation, open reduction, vastus medialis interposition, irreducible knee dislocation

## Abstract

Irreducible knee dislocation (KD) is a rare high-velocity injury (determined by shear and/or rotational forces) that is associated with the interposition of capsule-ligamentous or muscle structures in the joint space. This condition often requires open reduction. To our knowledge, irreducible KD is not widely reported in the literature. Here, we report the case of a 69-year-old man with a right KD that occurred after falling from a height and entrapment of the leg between concrete blocks. The patient presented to the emergency department with a right knee deformity without distal neurovascular deficit. After two failed attempts of close reduction (under sedation and spinal anesthesia), open reduction was performed. Intraoperatively, there were cruciate ligament tears, wide capsule tears, and intra-articular vastus medialis interposition, preventing reduction. The decision to release the muscle from the notch, suture of the medial capsule, temporary K-wire stabilization, and cast immobilization were taken. After K-wire removal, the patient underwent rehabilitation to regain function and resume activities of daily living. This case report highlights the need for open reduction in some KD cases. Identifying possible soft-tissue interposition can accelerate surgical treatment and minimize the risk of complications.

## Introduction

Knee dislocation (KD) is the disruption of contact between articular surfaces associated with multiple ligament injuries and multidirectional instability [1]. The disruption of the joint may cause multiple ligament injuries in at least two of the four paramount ligament structures, namely, anterior cruciate ligament (ACL), posterior cruciate ligament (PCL), posterolateral corner (PLC), and medial collateral ligament (MCL) [1].

The most used classification of KD based on the injured anatomical structure was described by Schnek. KD-I: ACL or PCL injury; KD-II: ACL and PCL only; KD-III: ACL, PCL, and posteromedial (PM) or posterolateral (PL) disruption; KD-IV: ACL, PCL, PM, and PL disruption; KD-V: KD and fracture association. The Schnek classification was modified by Yu who added the designation of C (arterial injury) and N (neural injury) [2].

Anterior KD is the most common type of injury caused by hyperextension of the knee, occurring in around 40% of the cases. Posterior KD (33%) is caused by posteriorly directed force to the tibia such as a dashboard injury. Valgus or varus loads can produce lateral (18%) or medial (4%) KD [1].

## Case presentation

A 69-year-old man presented to the emergency department (ED) with the chief complaint of right knee pain after falling from a height. He sustained a knee injury while falling from one and a half meters and entrapment of the right leg.

**Figure 1:**
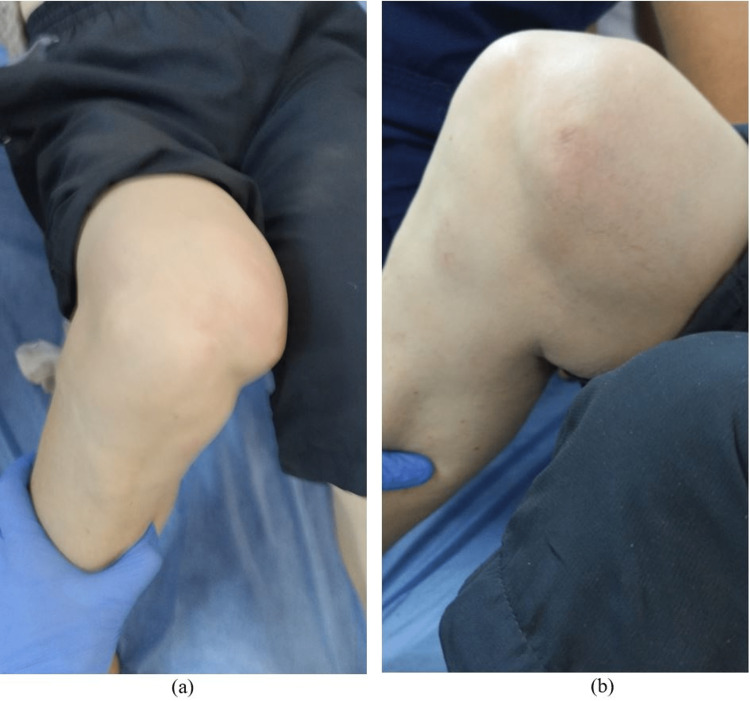
Physical examination of the right knee at the time of presentation. Valgus deformity and protrusion of the medial femoral condyle (1a) and at 90 degrees of knee flexion (1b).

Vital signs were normal, and the patient has no complaints aside from right knee pain. Initial inspection revealed an abnormal right knee contour characterized by a medial prominence above the knee, flexion, and valgus deviation of the knee (Figure 1).

**Figure 2:**
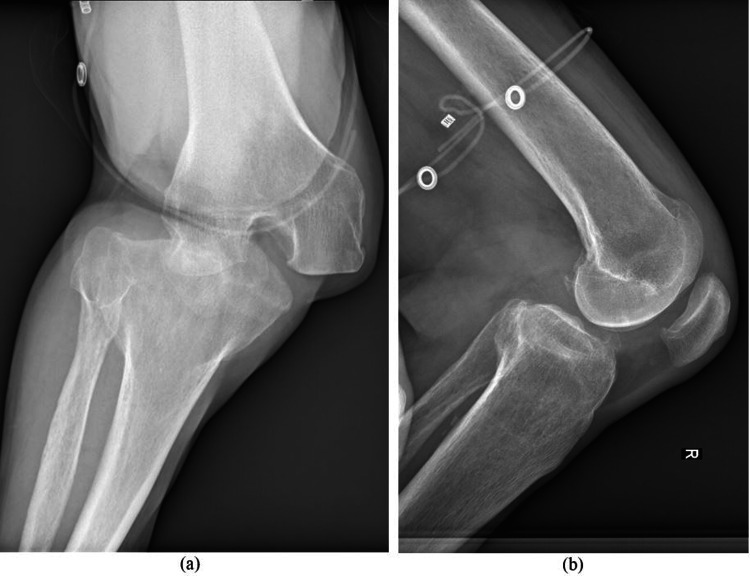
Anteroposterior and lateral radiographs of the right knee.

Physical examination showed palpable pedal pulses with intact sensation distal to the right knee. Computer tomography angiography was not done. Ligamentous stability and range of motion testing were not attempted due to patient discomfort. The patient stated that 40 years ago, he had suffered from knee dislocation of the same knee, which was treated by closed reduction and cast immobilization. After conservative treatment, the patient had a significant knee extension deficit (around 20 degrees). Before the injury, the patient had mild chronic pain in the right knee, a range of motion from 20 to 120 degrees, and on X-ray, there were femoral osteophytes and subchondral sclerosis (Figure 2).

The patient was limping and was wearing a heel pad on the right side to compensate for limb length discrepancy (around 25 mm). He was mobilizing with a sick. Plain radiographs confirmed the diagnosis of posterolateral KD without any fracture (Figure 2).

**Figure 3:**
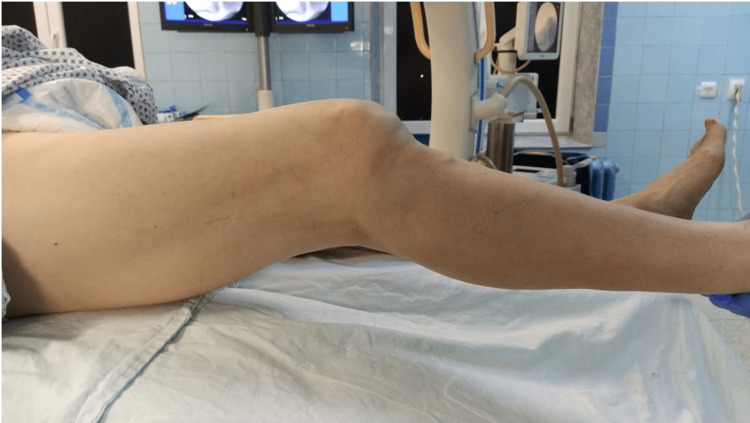
Physical examination after attempting a closed reduction in the operating theater. Preinjury alignment of the leg was achieved (~20 degrees of extension deficit and no valgus deformity).

**Figure 4:**
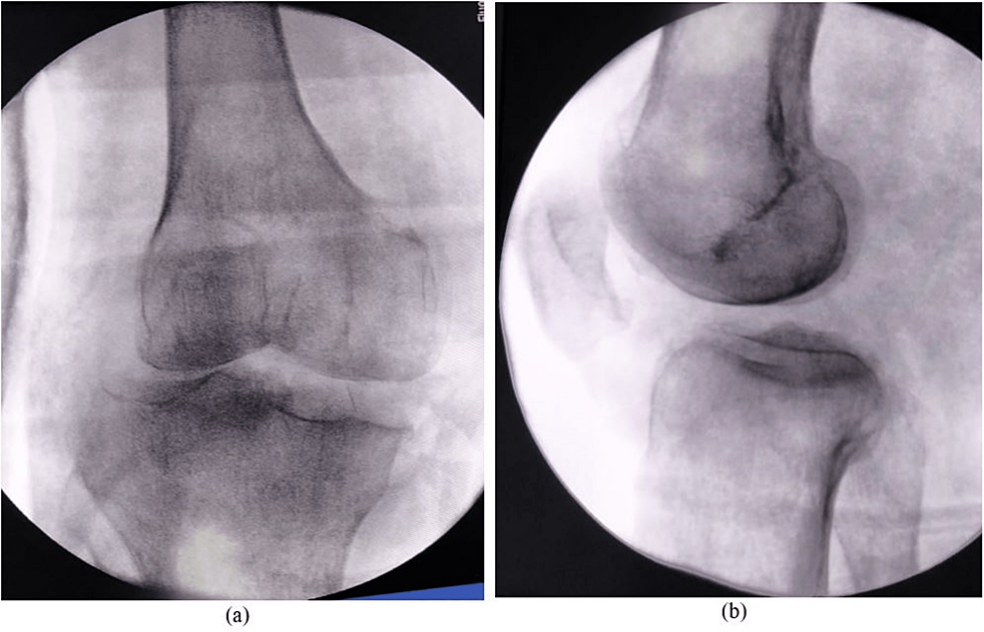
Anteroposterior and lateral radiographs showing posterolateral subluxation and medial tibiofemoral compartment widening.

Closed reduction under procedural sedation was attempted in the ED but was unsuccessful. As a reduction maneuver, traction and varus deviation were applied. An emergency close reduction was performed under spinal anesthesia in the operating room. Knee extension deficit (~20 degrees) (Figure 3), tibia subluxation, and widening of the medial tibiofemoral compartment were observed on C-arm radiographs (Figure 4).

**Figure 5:**
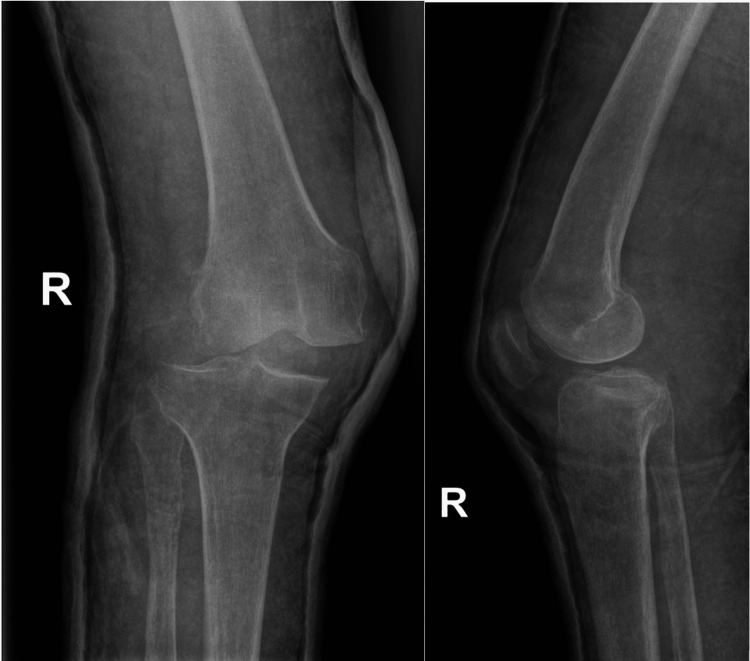
Anteroposterior and lateral view after close reduction of the right knee (in cast).

Popliteal and dorsalis pedis pulses were palpable. It was considered a satisfactory reduction because the patient was not able to fully straighten the knee before the injury, and cast immobilization was applied. The following day, a check X-ray was performed which showed valgus deformity and subluxated knee (Figure 5).

**Figure 6:**
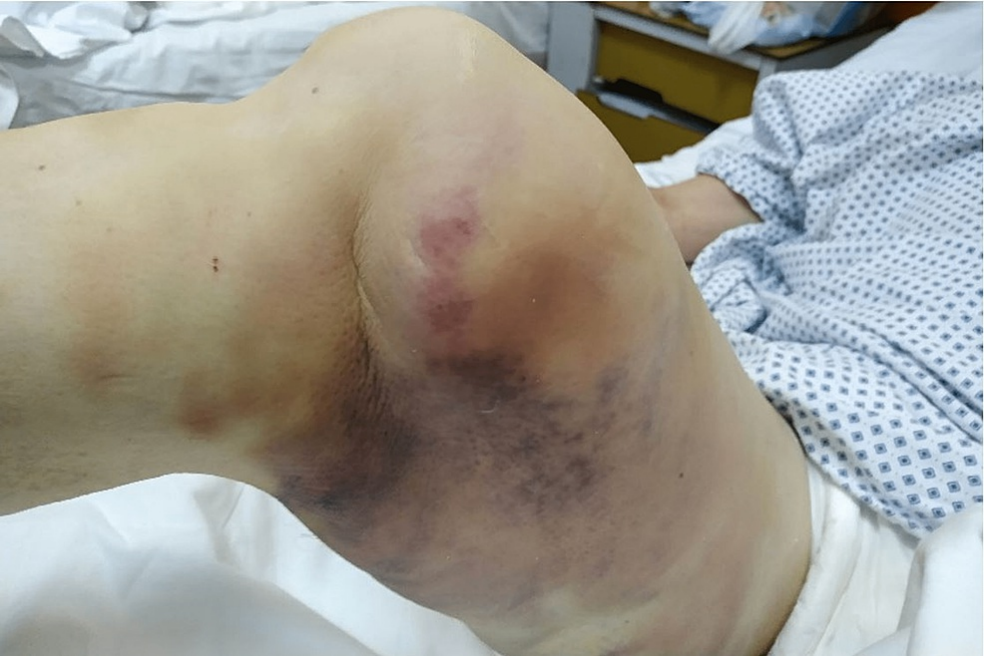
Skin pucker containing a recess at the level of the joint line, dimple sign, medial view.

**Figure 7:**
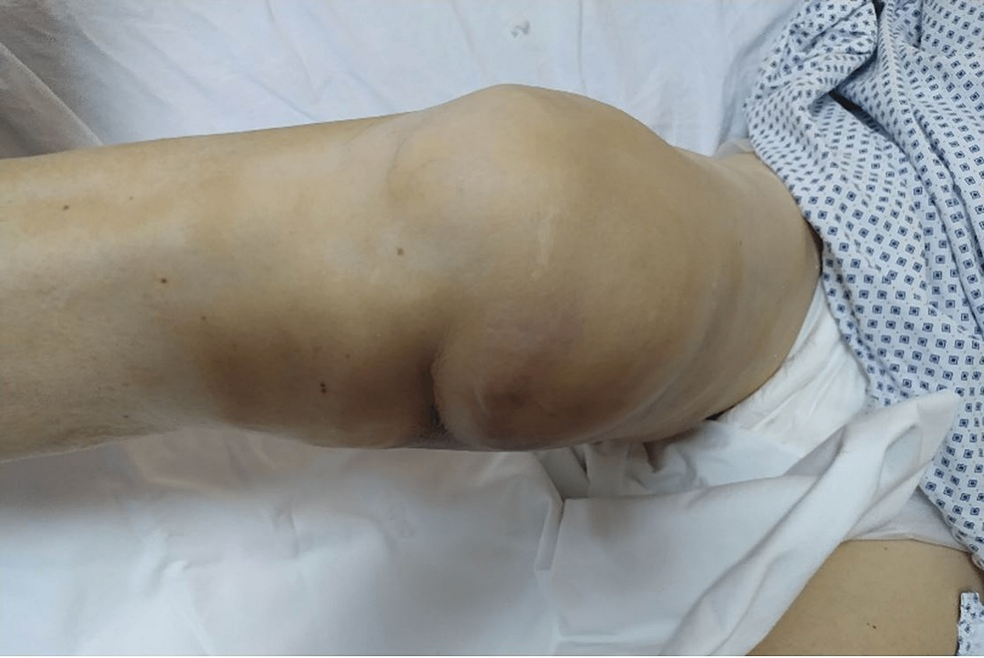
Skin pucker containing a recess at the level of the joint line, dimple sign, anterior view.

The cast was taken off, and a skin pucker with recess at the level of the joint was noted (Figures 6, 7).

**Figure 8:**
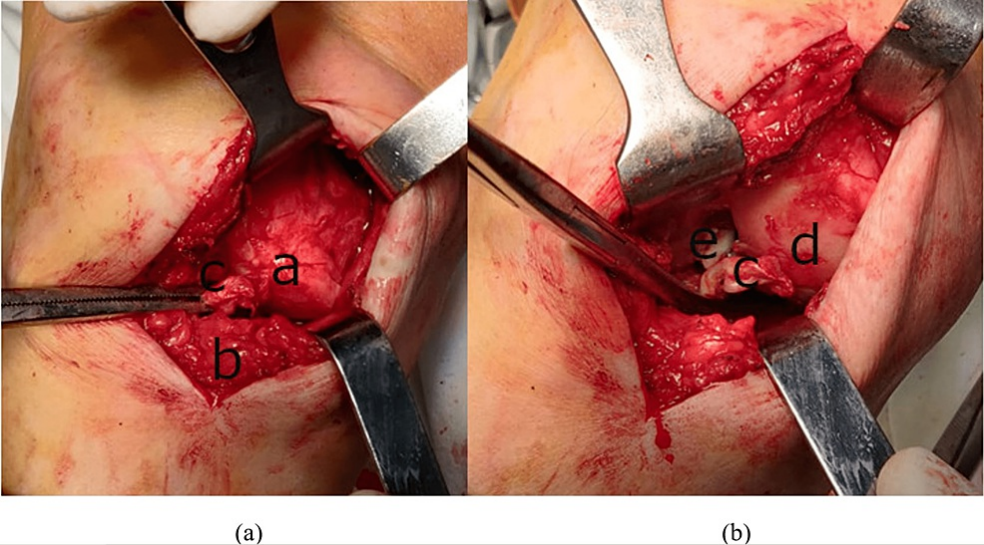
Intraoperative views demonstrating the buttonholded medial condyle. Before (a) and after (b) vastus medialis muscle release. Intraoperative views demonstrating the buttonholded medial condyle through the vastus medialis muscle belly. a: vastus medialis muscle belly; b: tibia; c: anterior cruciate ligament; d: medial femoral condyle; e: posterior cruciate ligament

It was considered failed reduction, and open reduction under spinal anesthesia was performed on the same day. A medial parapatellar approach was performed, and interposition of the vastus medialis muscle was observed. The midline approach was avoided because of the large incision. The distal portion of the muscle was dislocated inside the intra-articular space through the medial capsular lesion. Medial buttonholding of the femoral condyle and a tear in the medial capsule were observed. The incarcerated vastus medialis muscle belly was reduced from the notch and the medial femoral condyle (Figure 8a) using as a lever Cobb’s elevator. This restored the alignment and mobility of the joint and gave a better perspective of the capsular and ligamentous lesions (Figure 8b).

**Figure 9:**
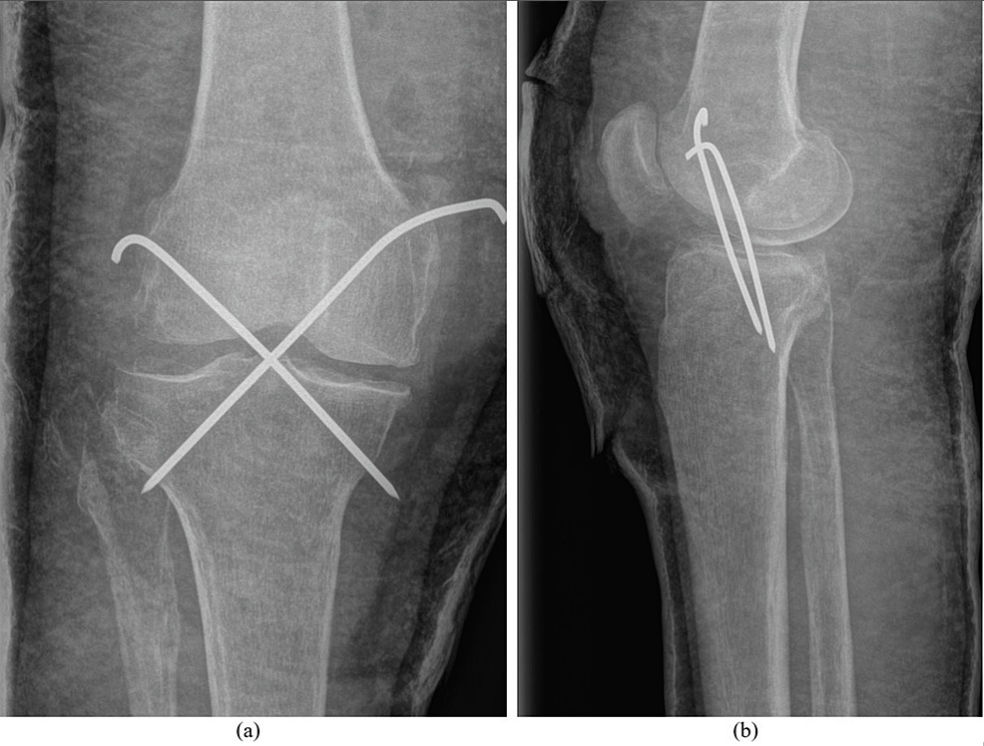
Temporary stabilization using K-wires and cast immobilization of the right knee, anteroposterior and lateral radiographs.

Both anterior and posterior cruciate ligaments were torn, and parrot beak tears of the medial meniscus and intact lateral meniscus were noted. A partial medial meniscectomy was performed. The range of motion was from 20 to 120 degrees, and the shortening of the lower limb was 25 mm. After reduction, knee stabilization was performed using two crossed 2.5 mm K-wires passed from the distal femur to the proximal tibia (Figure 9). The K-wires did not transfix the dislocation and cast immobilization was applied.

The knee joint was stabilized with a circular cast with an opening for wound dressing. As an anticoagulant therapy, the patient was administered subcutaneous nadroparin 0.4 mL every day for six weeks. The K-wires were removed after six weeks and the patient was sent for physiotherapy. An MRI examination of the knee prior to or after the removal of K-wires was not performed because the patient did not agree to be charged for the scan and there was no other facility. At the end of six months, the range of motion was from 20 degrees up to 100 degrees. The patient had the same limb length discrepancy and mild knee pain. Knee Society Clinical and Functional Score was 52/60.

Our plan is to perform a hinge total knee replacement if the patient continues to have significant pain in the future. The follow-up period of the patient was limited, as the patient did not attend the clinic 12 months after the injury.

## Discussion

KD is a rare [1] but serious injury with long-term implications. Most acute KD cases are reduced spontaneously at the accident site. The lesion of the popliteal artery is a severe complication of this injury which can result in ischemia, and, in some cases, can lead to amputation. Persistent ischemia longer than eight hours requires above-knee amputation in 86% of the cases [2]. Lateral dislocations can result in disruption of ACL, PCL, and MCL and can be associated with peroneal nerve injury. Associated fractures were diagnosed in 41.1% [3] of KD cases, and the incidence was higher in motor vehicle collisions but without distinguishing major articular fractures from ligament avulsion fractures.

Skin complications were reported in irreducible KD [4]. It can vary from a bruise to skin necrosis. The area of skin necrosis is usually limited [5,6].

The reduction of KD is usually simple [4], but some cases reported reduction before ED presentation [1]. Failure of closed reduction is rare [4]. Various causes have been described such as interposition of the patella [7], medial meniscus interposition [8], or buttonholding of the medial condyle through capsuloligamentous structures [4], which can make close reduction impossible.

The mechanism of injury of irreducible KD contains a rotational component [9]. In some cases, an association of internal rotation in irreducible KD has been demonstrated [10].

In acute complete KD, the ligaments should be repaired or reconstructed early followed by two weeks of cast immobilization because early ligament repair has been shown to have more satisfactory long-term results than cast immobilization alone [11]. Because of chronic knee injury followed by chronic knee pain and knee extension deficit, we performed open reduction and temporary arthrodesis. If significant chronic pain persists, we plan to offer total knee arthroplasty with a hinge prosthesis to the patient.

Although KD is a rare injury, irreducible KDs are much rarer and often reduced by open manipulation. This case underlines the necessity for early ligament repair in acute KD [11]. Further, femoral condyle buttonholding through a tear in the capsule has been rarely reported, and this injury requires open reduction. KD can be usually reduced by closed methods and around 50% are spontaneously reduced at the accident site [12]. Spontaneously reduced KDs prior to the clinical examination can make diagnosis difficult, and complications from the injury are easy to miss [13].

Complications are frequent and rarely does the knee return to a pre-injury state. Patients who are not selected for surgical repair usually have stiffness and loss of range of motion rather than instability as a long-term problem [14-16].

## Conclusions

Identifying complicated KD (soft-tissue interposition) during the physical examination and considering emergency open reduction are vital to minimize neurovascular damage. It is important to identify associated conditions such as osteoarthritis and chronic ligamentous injuries. For identifying multiple ligamentous injuries, an MRI examination is essential, especially for surgery planning.
